# Bionic Fluorine‐Free Multifunctional Photothermal Surface for Anti/de/Driving‐Icing and Droplet Manipulation

**DOI:** 10.1002/advs.202409631

**Published:** 2024-10-21

**Authors:** Lin Wang, Chengchun Zhang, Yasong Zhang, Chun Shen, Zhentao Xin, Zhenjiang Wei

**Affiliations:** ^1^ National Key Laboratory of Automotive Chassis Integration and Bionics Jilin University Changchun 130022 China; ^2^ College of Biological and Agricultural Engineering Jilin University Changchun 130022 China; ^3^ Weihai Junming Power Technology Co., Ltd Weihai 264200 China; ^4^ Institute for Bionics Jilin University Weihai 264402 China

**Keywords:** anti/de‐icing, bionic, droplet manipulation, fluorine‐free, photothermal

## Abstract

Due to safety concerns associated with ice accumulation, there is a need to develop a durable surface with anti/de‐icing properties. Inspired by Gecko skin and Nepenthes, a superhydrophobic photothermal surface (SH‐PS) with micro‐nano hierarchical structure based on carbon nanotube composites is prepared by one‐step laser method. It can be switchable to a slippery photothermal surface (S‐PS) by injecting silicone oil. S‐PS can effectively delay icing at −20 °C/−30 °C, and maintain excellent dynamic anti‐icing capabilities following 28 times supercooled droplet impact (−30 °C). Under near‐infrared (NIR) irradiation, S‐PS exhibits high‐efficiency photothermal deicing and excellent ice droplet control capabilities. Furthermore, S‐PS also exhibits remarkable droplet manipulation capabilities under NIR irradiation, even anti‐gravity transport (8°) and programmable track manipulation. Notably, after 20 m abrasion, the S‐PS still maintains good performances of anti/de‐icing and droplet manipulation after infusing lubricant. All these excellent performances can promote its application in a wider range of fields.

## Graphical abstract

A micro‐nano hierarchical superhydrophobic photothermal surface (SH‐PS) based on carbon nanotube composites is prepared by one‐step laser. Injecting lubricant turns it into a slippery photothermal surface (S‐PS), which has stable static/dynamic anti‐icing, photothermal deicing, and liquid/ice droplet control capabilities. Even abraded SH‐PS still presents remarkable anti/de/driving‐icing and droplet manipulation stabilities after infusing lubricant.

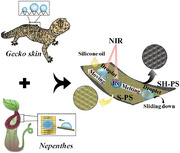

## 1 Introduction

The formation and buildup of ice can damage the operational efficiency of high‐speed trains, power lines, wind turbines, and aircrafts.^[^ ^1^, ^2^, ^3^, ^4^ ^]^ Traditional anti‐icing approaches, including antifreeze, thermal, mechanical, and chemical de‐icing methods, often are accompanied by elevated expenses, energy consumption, limited effectiveness, and unfriendly environment. Consequently, there is a necessity to devise more effective and environmentally sustainable methods to mitigate the adverse impacts of icing.

Superhydrophobic surfaces and slippery liquid‐injected porous surfaces (SLIPS) are highly valued for their exceptional hydrophobicity properties, such as rapid water removal, delayed icing, and low ice adhesion.^[^ ^5^, ^6^, ^7^, ^8^, ^9^, ^10^, ^11^, ^12^ ^]^ The repulsive force between water phase and air layer, water phase, and oil phase makes superhydrophobic surfaces and SLIPS exhibit excellent anti‐icing performance. However, due to the easy penetration of the air layer during prolonged exposure to low‐temperature environments, icing on the superhydrophobic surface is unavoidable.^[^ ^13^ ^]^ In this case, the ice is interlocked with the surface micro‐nano structure, significantly increasing ice adhesion.^[^ ^14^ ^]^ For SLIPS, the injection of lubricants can form a more stable lubricant interface rather than the air layer, and the ice‐pinning problem is well avoided.^[^ ^12^ ^]^ Nevertheless, when SLIPS is used in some actual harsh environments, it is inevitable that there will be ice water residue and gradual freezing, thus affecting its practical application. In contrast, photothermal surfaces made of carbon‐based materials exhibit the ability to perform photothermal deicing under NIR or sunlight illumination in low‐temperature conditions.^[^ ^15^, ^16^, ^17^, ^18^ ^]^ Sunlight is a clean and renewable energy source with wide applicability.^[^ ^19^, ^20^, ^21^ ^]^ However, it is easy to be limited by weather, uncontrollable light intensity, and direction. NIR has the advantages of adjusting light intensity and irradiation time, heating specific areas, and not being limited by weather. By combining slippery passive anti‐icing and photothermal active de‐icing technology, the limitations of traditional SLIPS in actual harsh icing environment are solved.^[^ ^22^ ^]^ Moreover, there are few reports on the study of photothermal driving ice water away from the surface directly and avoiding ice pinning. Nevertheless, the photothermal conversion efficiency of carbon‐based materials is usually relatively modest.^[^ ^23^, ^24^, ^25^, ^26^ ^]^ In order to enhance the photothermal conversion efficiency, adjusting the surface morphology of carbon‐based materials usually is used to generate more heat to melt ice on the surface within limited space.^[^ ^27^, ^28^, ^29^ ^]^ However, the preparation of photothermal anti‐icing surfaces with micro/nano structures involves complex preparation process, addition of toxic fluorine‐containing substances, and high cost. Additionally, the anti/de‐icing abilities of photothermal superhydrophobic surfaces are compromised or potentially eliminated when the micro‐nano structure is damaged, significantly limiting their application.^[^ ^1^, ^30^ ^]^ Hence, it is of great significance for practical applications to develop efficient and durable anti/de/driving‐icing surfaces using simple and environmentally friendly strategies.

As we all know, a dense array of micro and nano‐bumps of Gecko skin displays ultra‐low adhesion with droplets and contaminants, creating a superhydrophobic, anti‐wetting barrier.^[^ ^31^, ^32^, ^33^ ^]^ Additionally, the peristome of Nepenthes can become fully wetted by water, and then a slippery liquid film is formed to induce insects to fall toward the pitcher's mouth to meet their basic nutritional needs.^[^ ^34^ ^]^ Here, integrating the micro‐nano structure of Gecko skin with slippery of the Nepenthes, we fabricate a multi‐inspired slippery photothermal array based on carbon nanotube composites, which provides a feasible and effective avenue for anti/de/driving‐icing. At −30 °C, the S‐PS maintains excellent static and dynamic anti‐icing properties. Further, the dependability of the photothermal properties of S‐PS is also demonstrated by the cyclic light tests and the frozen droplets on them can be rapidly removed and even controlled under NIR. Furthermore, S‐PS displayed flexible manipulation in droplet mereging, microreaction, anti‐gravity manipulation, screening, and programmable control under NIR irradiation. More importantly, even after 200 abrasion cycles (20 m), surfaces still have good anti/de‐icing and droplet manipulation capabilities after infusing lubricant. This slippery strategy combining micro‐nano structure and photothermal property offers insights into the development of surfaces that integrated anti/de‐icing and droplet manipulation through a straightforward, environmentally friendly, and cost‐effective method.

## 2 Results and Discussion

### 2.1 Morphology and Wettability of Photothermal Surface

**Figure 1:**
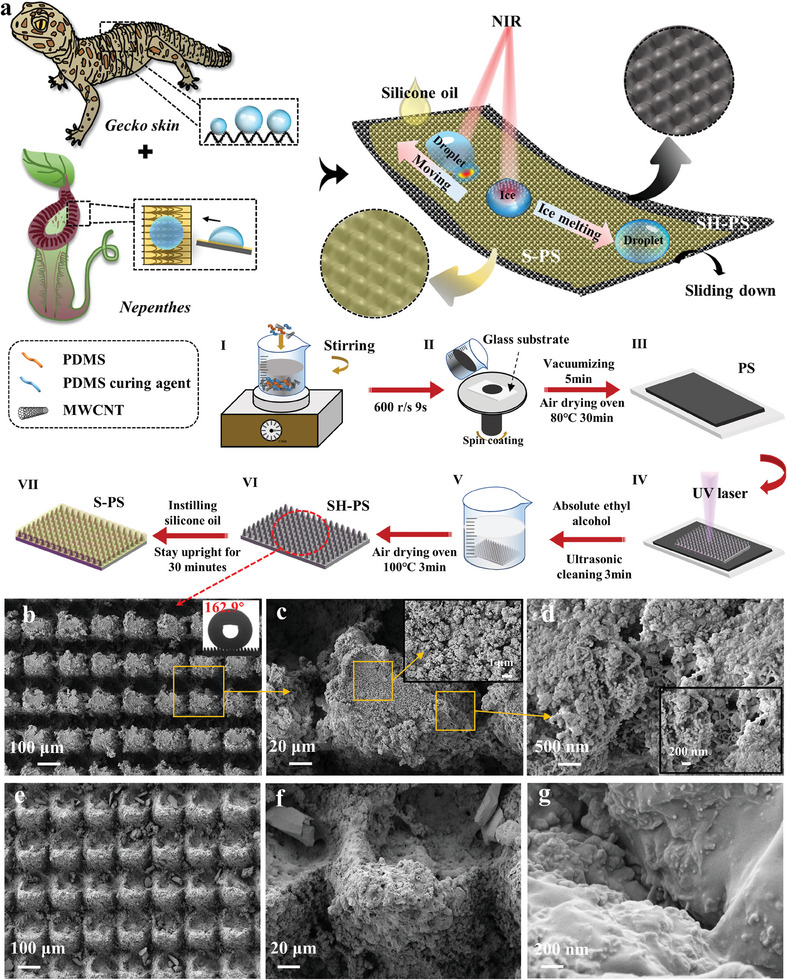
a) Schematic and preparation process of the S‐PS. SEM images of the SH‐PS (b–d) and SH‐PS after 200 abrasion cycles (e–g) at different scales.

Previous studies have indicated that micron‐scale and nano‐scale spinules are discernible on the skin of the gecko's dorsal and abdominal area.^[^ ^31^ ^]^ Inspired by the microstructure characteristics and superhydrophobic properties of gecko skin, the schematic and preparation process of SH‐PS is presented in **Figure** **1**a and Supporting Information. SH‐PS is comprised of a bionic microspine array with dimensions resembling gecko skin. Scanning electron microscope (SEM) (Figure 1b–d), energy dispersive spectroscopy (EDS) (Figure S1a, Supporting Information) and laser confocal scanning microscope (LCSM) (Figure S1b, Supporting Information) images reveal the micro‐nano rough porous structure of the SH‐PS, and the water contact angle (WCA) and sliding angle (SA) are 162.9° and 2°, respectively (Figure S1d, Supporting Information). The obvious affinity between SH‐PS and silicone oil and the porous rough micro‐nano structure is beneficial to the stable storage of silicone oil (Figure S1g, Supporting Information). When silicone oil is injected into SH‐PS, S‐PS shows slippery performance, similar to that of Nepenthes, and the WCA and SA reached 100° and 3°, respectively (Figure 1a; Figure S1e, Supporting Information). Generally, the wettability of surfaces following mechanical damage plays a significant role in their practical utility. To assess the mechanical durability of SH‐PS, sandpaper abrasion tests were conducted. Even after 200 times sandpaper abrasion (equivalent to 20 m abrasion distance, 6.67 kPa), the SH‐PS still possesses micro‐nano structure (Figure 1e–g) and exhibits excellent superhydrophobicity (Figure S1c, Supporting Information). In addition, the abraded S‐PS still maintains excellent slippery performance after the abraded SH‐PS was injected with silicone oil, and the SA is <5° (Figure S1f, Supporting Information).

### 2.2 Passive Anti‐Icing Performance

**Figure 2:**
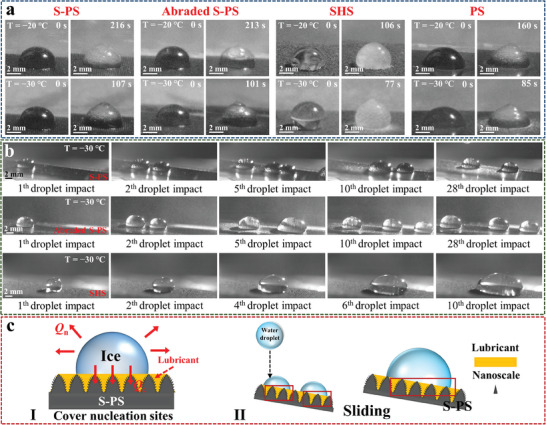
a) Static anti‐icing performance tests of S‐PS, abraded S‐PS, SHS, and PS surfaces at different temperatures. b) Dynamic anti‐icing property of S‐PS, abraded S‐PS, and SHS at −30 °C. High‐speed pictures show continuous droplets impacting on different supercooled surfaces (at 10° inclination). c) Schematics of heat transfer process (I) and dynamic anti‐icing (II) on S‐PS.

Experiments were performed to evaluate static anti‐icing performance under −20 and −30 °C ambient temperature, respectively. Different surfaces were positioned at −20 °C/−30 °C ambient temperature for 5 min to achieve a stable temperature. The freezing behavior and time of room temperature water droplets on various surfaces including S‐PS, abraded S‐PS, surface coated with commercial superhydrophobic mist spray (SHS), and the photothermal surface of PDMS with CNTs but without microstructure and silicone oil (PS) are observed at controlled environmental temperatures (**Figure** **2**a). Results indicate that at −20 °C (−30 °C), the time required for complete freezing of water droplets on S‐PS, abraded S‐PS, SHS, and PS is 216 s (107 s), 213 s (101s), 106 s (77 s), and 160 s (85 s), respectively. S‐PS exhibits the longest delayed icing time among the surfaces tested, indicating superior anti‐icing capabilities (Table S1, Supporting Information). Notably, even the abraded S‐PS exhibits better icing delay performances than SHS and PS.

Further, the droplet freezing processes on the surface are elucidated through heat transfer models (Figure 2cI). Regarding slippery surfaces, this heat transfer process mainly involves conduction heat loss (*Q*_c_) and the convection heat loss (*Q*_n_).^[^ ^35^ ^]^ The heat loss (Δ*Q*) mechanism resembles the Wenzel state, described as:

(1) $$\Delta Q=Q_{c}+Q_{n}$$

*Q*_c_ represents the primary form of droplet heat loss during icing delay. To quantify droplet heat loss on different surfaces during icing process, the heat loss rate (*η*) of water droplets on the respective surface is defined as:^[^ ^36^, ^37^ ^]^

(2) $$\eta =\frac{\Delta Q}{T}$$

Δ*Q* is the water droplet heat loss during icing process, and *T* is the water droplet freezing delay time.

For SHS, the existence of the air layer reduces the heat transfer between the metal surface and the droplets by reducing the contact area between the substrate and the droplets. However, at low temperatures, the air layer is easily lost, and water droplet will easily adsorb on the surface wall and freeze. PS hinders the heat exchange between the cold substrate and the droplet through the film and prolongs the freezing time. Compared with SHS and PS, the delayed freezing time of S‐PS and abraded S‐PS increased significantly. The main reason is that the surface of S‐PS before and after abrasion is replaced by liquid–liquid contact instead of gas–liquid contact or solid‐liquid contact. The lubricating layer forms a thermal barrier between the droplet and the cold surface, which effectively reduces the heat loss of the droplet, limits the heat transfer, and increases the icing time.^[^ ^38^, ^39^ ^]^ Therefore, compared with SHS and PS, S‐PS and abraded S‐PS exhibit lower *Q*_c_. Moreover, S‐PS and abraded S‐PS have higher *T*, which indicates that S‐PS and abraded S‐PS have smaller *η*. The results show that the porous microarray structure injected with silicone oil can effectively prolong the freezing time of water droplets, making it widely used in the field of anti‐icing.

In practical scenarios, surface icing is primarily induced by supercooled raindrops (0 °C) and environments.^[^ ^40^, ^41^ ^]^ To simulate the practical situations of supercooled water droplet striking the surface in extremely low temperature condition, the dynamic anti‐icing tests were conducted (Figure 2b; Movie S1, Supporting Information). At −20 °C, when the first water droplet contacts with the supercooled surface of SHS, the water droplet rebounds, leaving small needle‐like remnants of water droplets. Subsequent water droplets adhere to the SHS surface, gradually accumulating and nucleating to form ice crystals as a result of continuous water droplet impact. At −30 °C, the pinning phenomenon is observed upon the first water droplet impacts on the SHS. Despite the SHS having an inclination angle of 10°, after ten consecutive droplet impacts, it becomes coated with accumulated ice‐water mixture. In contrast, silicone oil filling in micro‐gaps on the S‐PS surface functions similarly to air pockets, and the presence of silicone oil on the S‐PS surface leads to complete repulsion of water droplets during impact due to sliding at the liquid‐liquid interface(Figure 2b,cII).^[^ ^42^ ^]^ The low surface friction of silicone oil allows water droplets to slide along the S‐PS surface with less energy loss compared to a bare surface. Moreover, even abraded S‐PS still successfully slide down droplets despite being repeatedly impacted by supercooled water droplets 28 times, which is similar to the original S‐PS dynamic anti‐icing ability. The results highlight that S‐PS demonstrates the best anti‐icing performance compared to the other surfaces.

### 2.3 Active de‐Icing Performance

**Figure 3:**
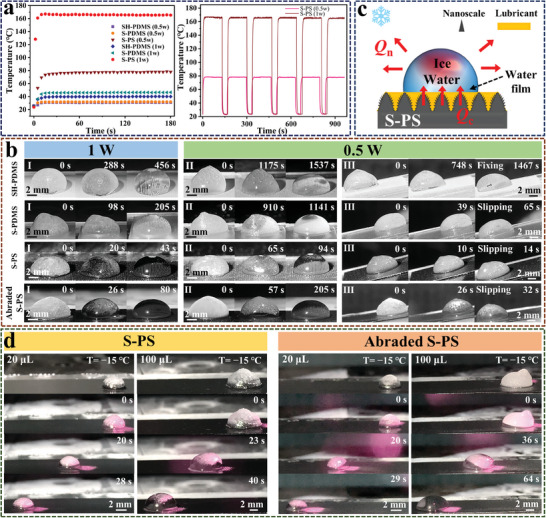
a) The surface temperature variation of various samples (SH‐PDMS, S‐PDMS, and S‐PS) under different power NIR irradiation (Left). The temperature variation of S‐PS during 6 repeated ON/OFF NIR irradiation cycles under different power NIR irradiation (Right). b) The photothermal de‐icing process of 100 µL frozen water droplet on SH‐PDMS, S‐PDMS, S‐PS, and abraded S‐PS surfaces at 1 W (I) and 0.5 W (II) irradiation intensities (at −15 °C environmental temperature). (III) Photothermal de‐icing process of 100 µL freezing droplet on inclined (*θ* = 15°) various surfaces under 0.5 W NIR irradiation. c) Illustration of the heat transfer process between frozen droplets and surfaces under NIR irradiation. d) Different scales of ice droplets are driven by 1 W NIR irradiation at −15 °C.

Although the S‐PS surface has excellent anti‐icing performance, there will still be ice water residue and reduced anti‐icing effect under extreme environmental conditions. Hence, it is crucial to endow the de‐icing property to materials.^[^ ^27^, ^43^, ^44^ ^]^ The S‐PS surface demonstrates excellent photothermal conversion ability. The temperature change of different surfaces with time under 0.5/1 W irradiation intensities is shown in **Figure** **3**a, and the initial surface temperature is 20 °C. During the identical NIR irradiation time, the temperatures of S‐PS surfaces are two times higher than SH‐PDMS and S‐PDMS. Particularly, as the NIR output power is increased to 1 W, the temperatures of S‐PS surfaces increase to >160 °C. Moreover, when S‐PS is placed in an oven at 160 °C for 5 h, it still maintains excellent slippery performance (SA < 10°) (Figure S2, Supporting Information). The strong affinity of PDMS and silicone oil and the porous rough micro‐nano structure make S‐PS maintain excellent stability under NIR of 1 W.^[^ ^22^ ^]^ Furthermore, after six repeated ON / OFF NIR irradiation cycles, the heat generation capacity of the S‐PS is well maintained. This suggests that their photothermal performance is stable and suitable for long‐term application (Figure 3a).

Through a comparative analysis of surface temperatures, it can be determined that surfaces incorporating CNTs exhibit remarkable photothermal conversion capacity. The excellent photothermal conversion of S‐PS can be attributed to the following aspects. First, the inherent blackbody characteristics of carbon‐based materials endow them with photothermal conversion capabilities. Second, the micro‐nano hierarchical structure can enhance the light absorption through the light capture effect. The light is captured in the hierarchical micro‐nano structure, resulting in multiple internal reflections, thereby improving the photothermal conversion efficiency.^[^ ^27^, ^45^ ^]^ Further studies evaluated the effectiveness of various surfaces in photothermal de‐icing. The process of ice melting on four different surfaces under varying irradiation intensities is depicted in Figure 3b. The completely frozen peach‐shaped ice droplets begin to melt first at the interface between the droplet and substrate across all samples under NIR irradiation. To be specific, at an irradiation intensity of 0.5 W (1 W), the droplets completely melt in 1537 s (456 s), 1141 s (205 s), 94 s (43 s), and 205 s (80 s) on SH‐PDMS, S‐PDMS, S‐PS, and abraded S‐PS, respectively. Evidently, with the increase of irradiation intensity, there is a notable decrease in the de‐icing time. It is worth noting that the photothermal de‐icing time of SH‐PDMS surface under 0.5 W infrared light irradiation is 16 times and seven times that of S‐PS and abraded S‐PS, respectively. These results can determine that the photothermal de‐icing time of S‐PS and abraded S‐PS are notably lower than that of the surface without CNTs. The abraded S‐PS remained stable de‐icing performance, and the de‐icing time is about twice that of the original S‐PS's photothermal de‐icing time. The results show that the presence of CNTs is particularly critical for photothermal de‐icing. Due to the uniform mixing of CNTs and PDMS, CNTs are uniformly distributed inside SH‐PS. After 20 m abrasion, the internal distribution of CNTs can be observed in SH‐PS (Figure 1e–g), so that it still maintains good de‐icing performance after injecting lubricant.

The mechanism of this photothermal de‐icing process is shown in Figure 3c. For the S‐PS surface, the deposited silicone oil displaces the air cavitations, which are supported by the hierarchical structure. The latent heat (*Q*_s_) released can then be conveyed to the water droplet. Thus, the heat transfer Equation on the slippery surface is formulated as:^[^ ^43^, ^46^, ^47^ ^]^

(3) $$\Delta Q=Q_{c}-Q_{n}$$

Δ*Q* represents the thermal gain of the droplet in unit time. Under NIR irradiation conditions, the droplet benefits from the effective photothermal characteristics of S‐PS, enabling it to progressively absorb heat through conduction (*Q*_c_). Meanwhile, the droplet experiences heat loss through natural convection (*Q*_n_).

Although the time required to melt ice on S‐PS surface is shorter, the primary objective in deicing is the swift removal of ice from the surface rather than complete melting. In Figure 3bIII, the experimental surface is placed in a cold chamber at a 15° incline angle. Upon subjecting the surface to a temperature of −15 °C and applying 0.5 W NIR irradiation, the ice droplet on the SH‐PDMS surface completely melts within 1467 s, but remains affixed to the surface. In contrast, the ice droplets on the S‐PDMS, S‐PS, and abraded S‐PS surface start to slide after 39, 10, and 26 s slid down due to their lubricant, respectively. Consequently, it proves that the S‐PS surface in both states possesses good anti‐icing and de‐icing properties. Additionally, by studying photothermal deicing process, it is found that Marangoni‐induced ice droplet self‐moving occurs in which ice droplets move directionally under the 1 W NIR irradiation and gradually melt (Figure 3d; Movie S2, Supporting Information). This phenomenon is a result of the movement of bubbles within ice droplets, wherein the bubbles are driven by Marangoni forces from areas of low temperature without irradiation to areas of high temperature with irradiation, thus facilitating the motion of the ice droplets (Figure S3, Supporting Information).^[^ ^48^ ^]^

### 2.4 Droplet Manipulation

**Figure 4:**
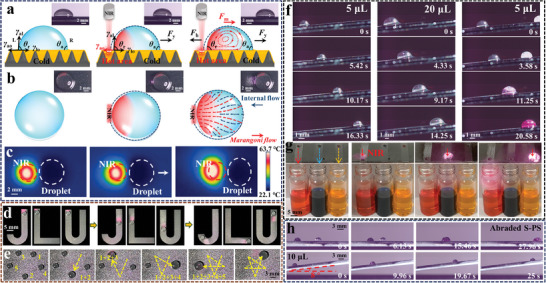
Schematic and photo images inside (a) and top (b) views of the deionized water droplet (100 µL) motion on the S‐PS by NIR actuation. c) The IR images in top view of the deionized water droplet (100 µL) motion on the S‐PS by NIR actuation (0.5 W). d) Programmable droplet manipulations on S‐PS. e) Images of light‐induced droplet coalescence in sequence on S‐PS. f) NIR‐induced antigravity motion and coalescence of droplets on 8° inclined S‐PS. Left: 5 µL water droplet; Center: 20 µL water droplet; Right: condensation of 5 µL NaOH droplet and 5 µL phenolphthalein droplet. g) Droplet screening with designed pathways. h) NIR‐induced coalescence and antigravity motion of 10 µL droplets on abraded S‐PS.

Apart from the mentioned above, S‐PS also exhibits considerable potential applications in droplet manipulation due to the synergistic effect of good slippery and photothermal properties (**Figure** **4**; Figure S4, Supporting Information). Under an uneven light source, the temperatures on both sides of the droplet changed. When the spot center is applied to the left side of the droplet, the temperature on the left side is higher than that on the right side. The temperature gradient will induce Marangoni flow in the droplet, generating a driving force to manipulate the droplet.^[^ ^49^, ^50^, ^51^, ^52^ ^]^ The flow generation sequence is observed from a side view, where surface shear flow initiates from the heated region, leading to internal flow within the droplet (Figure 4a; Movie S3, Supporting Information). From a top view, the left side of the droplet visibly contracts upon NIR irradiation, ultimately causing the droplet to move toward the opposite side as a result of the different surface tension on both sides (Figure 4b). The directional motion of the water droplet is indicated by arrows in Figure 4a,b. According to wettability theory, the apparent contact angle (*θ*) of a stationary droplet on surface can be described using Young's eqaution:^[^ ^53^ ^]^

(4) $$\gamma_{\mathrm{al}}\cos\theta =\gamma_{\mathrm{ao}}-\gamma_{\mathrm{lo}}$$

The tensions at the interfaces between air‐liquid, air‐oil, and liquid‐oil are denoted as γ_al_, γ_ao_, and γ_lo_, respectively. Contact angles are specified at two different locations on the droplet: the receding contact angle (*θ*_r_) on the left side where NIR action occurs (Left‐L), and the advancing contact angle (*θ*_a_) without NIR influence (Right‐R). When NIR is applied, a dynamic temperature gradient is generated on the irradiated section of S‐PS. As per Equation (4), when the dynamic temperature gradient reaches side L of the droplet, the surface tension of the oil–air interface at side L of droplet (*γ*_ao_(L)) decreases, leading to an increase in *θ*_r_ and disrupting the equilibrium of contact angles on the droplet. This asymmetric deformation (*θ*_r_ > *θ*_a_) of the droplet can be sustained during droplet movement and results in an imbalanced Young's force (*F*_y_) acting on opposite sides of droplet. *r* represents the droplet fundamental radius. *F*_y_ can be provided as follows:^[^ ^49^, ^54^, ^55^, ^56^ ^]^

(5) $$F_{y}=2r\left(\cos\theta_{a}-\cos\theta_{r}\right)\gamma_{\mathrm{al}}$$

Moreover, as a result of thermal exchange between the S‐PS and droplet, a significant temperature gradient is formed within the side L of the droplet. The spatial inconsistency in surface tension due to this temperature variation leads to the generation of additional tangential stresses known as Marangoni force (*F*_m_). It can be described as follows:^[^ ^49^, ^50^, ^57^, ^58^, ^59^ ^]^

(6) $$F_{m}\approx \pi r^{2}\frac{d\gamma_{\mathrm{al}}}{dT}\frac{dT}{dx}$$

The *F*_m_ points toward the side of the droplet with low‐temperature side in the *x*‐direction. *dγ*_al_ */dT* represents change in interfacial tensions between liquid and air as a function of temperature. *dT/dx* signifies the thermal gradient along the droplet surface in the *x*‐direction, where *x* denotes the droplet movement direction.

In general, the hydrodynamic resistance (*F*_h_) acts in the opposite direction to the motion tendency of the droplet. It includes the viscous forces within droplet and the wetting ridge formed at the base of the droplet on surfaces infused with oil. It is characterized by follows:^[^ ^49^, ^50^, ^53^, ^58^ ^]^

(7) $$F_{h}\propto \left(\mu_{o}+\mu_{l}\right)v\pi r$$

where *µ*_o_ denotes the oil viscosity, *µ*_l_ represents the liquid viscosity, and *v* indicates the droplet velocity. It can be observed from Equation (7) that *F*_h_ will rise as the droplet velocity increases. By considering Equations (5)–(7), it is observed that during the initial phase of light‐induced droplet motion, the combined forces of *F*_m_ + *F*_y_ > *F*_h_, result in droplet acceleration. Once *F*_m_ + *F*_y_ = *F*_h_, the droplet will move at a constant speed.

Furthermore, the infrared images depicting a droplet, outlined by white dashed circles, being transported on S‐PS triggered by NIR irradiation, indicated by red dashed circles, are given in Figure 4c. When near‐infrared radiation acts on the side of the droplet, the droplet commences its movement. The color spots signify the photothermal response on S‐PS, indicating that a significant temperature gradient is formed at the irradiated area of S‐PS.^[^ ^49^ ^]^ Moreover, the impacts of both NIR power and droplet volume on the velocity of droplet also are investigated. Usually, for the same volume of water droplets, higher power NIR will lead to a higher heating rate.^[^ ^50^ ^]^ Therefore, the velocity of droplet rises in correlation with the increase in NIR power. For a given 0.5 W NIR power, smaller droplets exhibit faster movement than larger droplets. Hence, it can be inferred that droplets of smaller volume achieve higher velocities under the same power (Figure S5, Supporting Information).

In practical applications, the ability to control droplet movement along desirable paths is crucial.^[^ ^60^, ^61^, ^62^ ^]^ In this work, S‐PS exhibits programmable droplet manipulation abilities on various paths under NIR irradiation. Droplets are controlled to follow designed paths, such as “J”, “L”, and “U” (Figure 4d; Movie S4, Supporting Information). Besides, through the manipulation of the irradiation position of NIR along the predetermined trajectories, such as “pentagram”, water droplets could merge sequentially (Figure 4e; Movie S4, Supporting Information). Significantly, as shown in Figure 4f (Movie S5, Supporting Information), the antigravity transportation of droplets (5–20 µL) is achieved on the 8° inclined S‐PS through NIR irradiation. In addition, a NaOH droplet is propelled uphill by NIR on S‐PS, while a phenolphthalein droplet moves downward under the influence of gravity. Once coalesced, the combined droplet can continue to move upward. Moreover, the precise distribution of droplets to desired locations without cross‐contamination is achievable, facilitating droplet screening with predefined pathways (Figure 4g). Notably, abraded SH‐PS retains the capacity for various forms of droplet manipulation by injecting silicone oil (Figure 4h; Movie S6, Supporting Information). In conclusion, S‐PS has the multi‐functional intelligent control ability of droplets.

## 3 Conclusion

In summary, we have prepared S‐PS with great slippery and photothermal performance by infusing silicone oil into micro‐nano hierarchical structures utilizing a simple, fluoride‐free, and cost‐effective strategy. S‐PS could efficiently delay the icing of static droplet and maintain stable dynamic anti‐icing properties after supercooled droplet impact. Moreover, under NIR irradiation, S‐PS possesses high‐efficiency photothermal deicing and manipulates ice droplet capability. More importantly, the abraded SH‐PS surface exhibits remarkable anti/de/control‐icing and droplet manipulation stability after infusing lubricant. Furthermore, the S‐PS also demonstrates exceptional capabilities in multifunctional droplet manipulation under NIR irradiation, which can realize horizontal droplet movement, merging and microchemical reaction, anti‐gravity manipulation (8°), and even programmable droplet track manipulation. The S‐PS with anti‐icing and photothermal deicing characteristics holds potential for applications in industrial anti‐icing. Additionally, it offers a possible avenue for the advancement of integrated multifunctional surfaces for anti/de/driving‐icing and droplet manipulation.

## Conflict of Interest

The authors declare no conflict of interest.

## Supporting information



Supporting Information

Supplemental Movie 1

Supplemental Movie 2

Supplemental Movie 3

Supplemental Movie 4

Supplemental Movie 5

Supplemental Movie 6

## Data Availability

Research data are not shared.
