# Supplementary material for: Bionic Fluorine‐Free Multifunctional Photothermal Surface for Anti/de/Driving‐Icing and Droplet Manipulation
Source: Adv Sci (Weinh). 2024 Oct 21;11(46):2409631. doi: 10.1002/advs.202409631 (PMC11633505; doi:10.1002/advs.202409631)
Supplement: Supplementary file 1 — Supporting Information [file ADVS-11-2409631-s006.docx]

Supporting information

**Bionic fluorine-free multifunctional photothermal surface for anti/de/driving-icing and droplet manipulation**

*Lin Wang, Chengchun Zhang^*^, Yasong Zhang, Chun Shen, Zhentao Xin, Zhenjiang Wei*

**1. Experimental Section**

*Materials*: Carbon nanotubes (CNTs, 20 nm) were provided by Suzhou Graphene Technology Corporation. Polydimethylsiloxane (PDMS, Sylgard 184) and curing agent of it were purchased from Dow Corning Co., Ltd. Silicone oil (50 cs) was bought from DOW (ZHANGJIAGANG) Holding Co., Ltd. Absolute ethyl alcohol was supplied by Sinopharm Chemical Reagent Corporation.

*The preparation of* *photothermal surface*: CNTs, PDMS, and a curing agent were combined in a weight proportion of 0.5:10:1 and mechanically mixed for 10 minutes at a speed of 2500 rpm. 1 g composite mixture was then poured onto a glass slide and spin-coated for 18 s at 1000 rpm. Subsequently, the glass slide was positioned within a vacuum chamber for 10 min to eliminate bubbles, and then cured at 80 °C for 30 min. Ultimately, the cured composites of CNTs and PDMS were photothermal surface (PS) (Figure 1a).

*The preparation of superhydrophobic photothermal surface and* *slippery photothermal surface*: The PS underwent 5 times of etching using UV laser marking machine (UV-50, China) and ultrasonically cleaned with anhydrous ethanol to obtain a superhydrophobic photothermal surface (SH-PS) with a diameter of 100 µm, a height of 150 µm and a spacing of 50 µm. Then, the SH-PS was immersed in 1cSt (50) silicone oil for 12 h. Following this, it was removed from the container and positioned in an upright orientation for 10 min to eliminate any surplus silicone oil. At last, a slippery photothermal surface (S-PS) was successfully obtained (Figure 1a).

*Characterization*: The structure and chemical makeup of samples were analyzed by field emission scanning electron microscope (FESEM, Zeiss EVO 18, Germany). Surface topography was assessed through three-dimensional (3D) imaging with a laser confocal scanning microscope (LCSM, OLS3000). Optical videos and photographs were captured using high-speed camera (Phantom v711).

*Static and dynamic anti-icing test*: The static and dynamic anti-icing performances of surface coated by commercial superhydrophobic mist spray (SHS), raw photothermal surface (PS), S-PS and abraded S-PS were investigated by a specially designed apparatus. For the static anti-icing test, initially, different surfaces were positioned at −20 °C/−30 °C ambient temperature for 5 min to achieve a stable temperature. Approximately 30 μL of water droplets at room temperature were subsequently placed horizontally on each supercooled sample, and times it took for the water droplets to freeze were measured. The freezing process was documented using a camera.

Experiments were conducted to test the dynamic anti-icing properties of various samples under ambient temperatures of −30°C. SHS, S-PS, and abraded S-PS were positioned in the freezing apparatus at a 10 ° inclination angle, and water droplets of approximately 30 μL (0 °C) were released from a height of 1-2 cm to impact samples. The dynamic anti-icing behavior of samples was captured by a high-speed camera.

*De-icing test*: An infrared thermal imager was used to record surface temperature and infrared thermal imaging images under a near-infrared laser (808 nm, 0.5 W/1 W, SZ laser) to assess photothermal characteristics. Furthermore, the photothermal de-icing capabilities were evaluated by the measurement of the time required for ice to melt on the sample surfaces. De-icing trials were conducted within a customized freezing chamber. 100 μL water droplets were respectively placed on SH-PDMS (without CNTs), S-PDMS (without CNTs but slippery), S-PS and abraded S-PS surface for freezing at −15 °C in the customized device. After 6 h of freezing, ice droplets were formed on the surface. The samples were illuminated by infrared light for the deicing experiment. The process of complete melting of ice droplets and and time required were documented using a video camera.

*Sandpaper abrasion*: To assess the mechanical durability of SH-PS, sandpaper abrasion tests were conducted. The SH-PS was abraded using sandpaper with a grit size of 800, and a weight of 200 g was applied to it. A linear motion of 10 cm on sandpaper is defined as a cycle.

**2. Supplementary Figures**


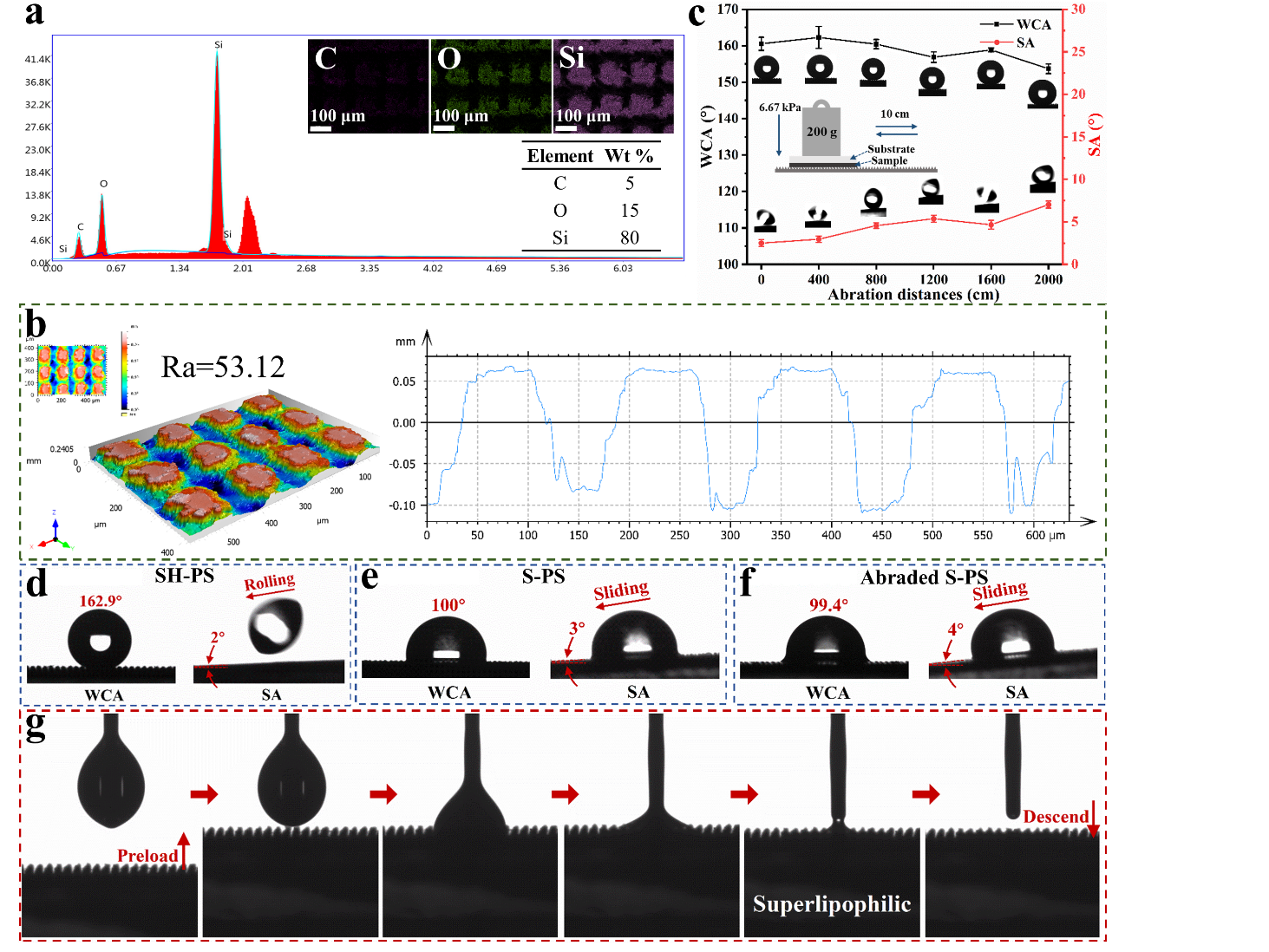


**Figure S1.** (a) EDS and (b) LCSM images of the SH-PS. (c) Sandpaper abrasion schematic diagram and the variation of water contact angle (WCA) and sliding angle (SA) after 200 times sandpaper abrasion tests for SH-PS. WCA and SA characterization of the prepared (d) SH-PS, (e) S-PS and (f) abraded S-PS. (g) The characterization of the affinity of the SH-PS surface for silicone oil.


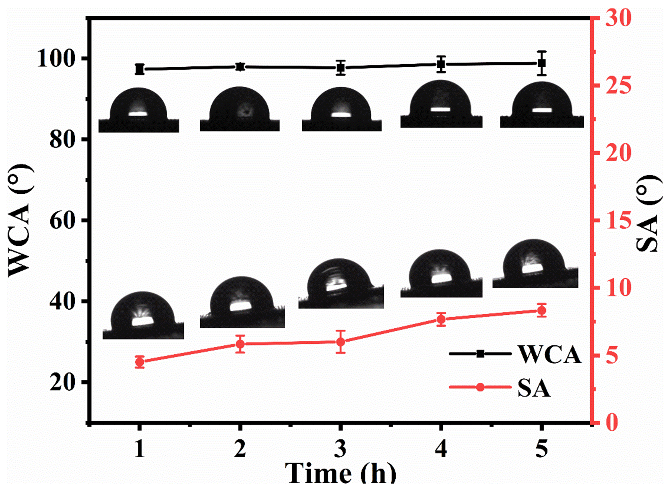


**Figure S2.** The variation of WCA and SA of S-PS during being placed in an oven at 160 °C for 5 h.


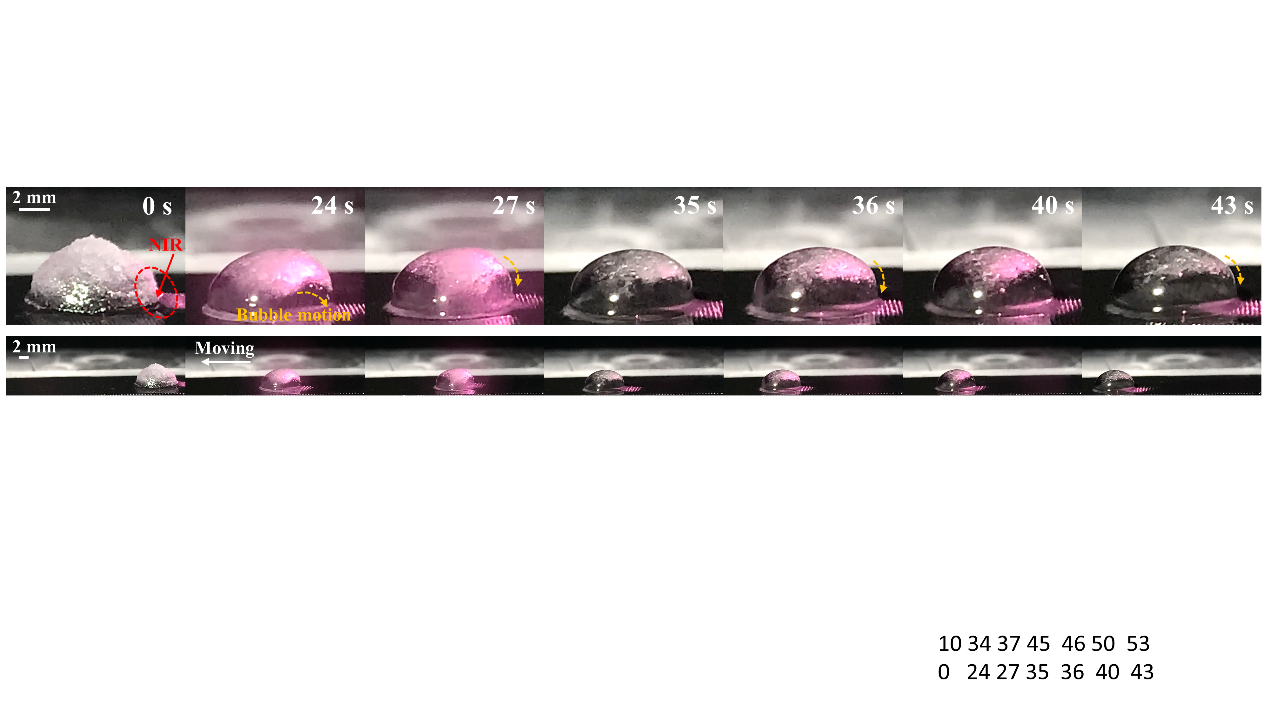


**Figure S3.** 100 μL ice droplet on S-PS are driven by 1 W NIR irradiation at −15 ℃.


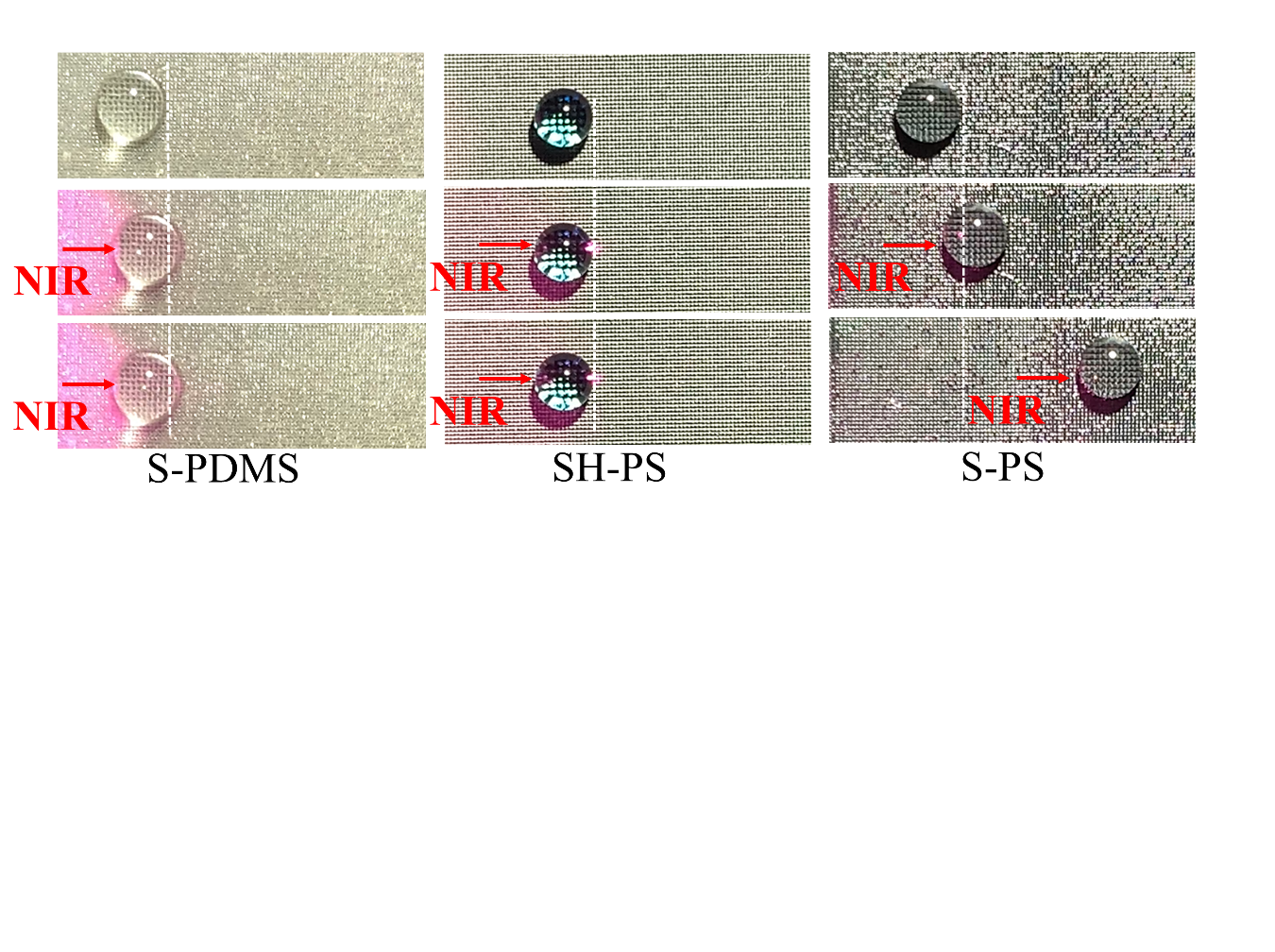


**Figure S4.** The states of a water droplet (~5 μL) on the S-PDMS, SH-PS and S-PS under long term NIR irradiation. Control tests were performed on S-PDMS (sample lacking CNTs, no photothermal performance compared to S-PS) and SH-PS (sample without oil infusion, lacking low hysteresis compared to S-PS). Light-induced droplet motion was not observed on S-PDMS or SH-PS but on S-PS.


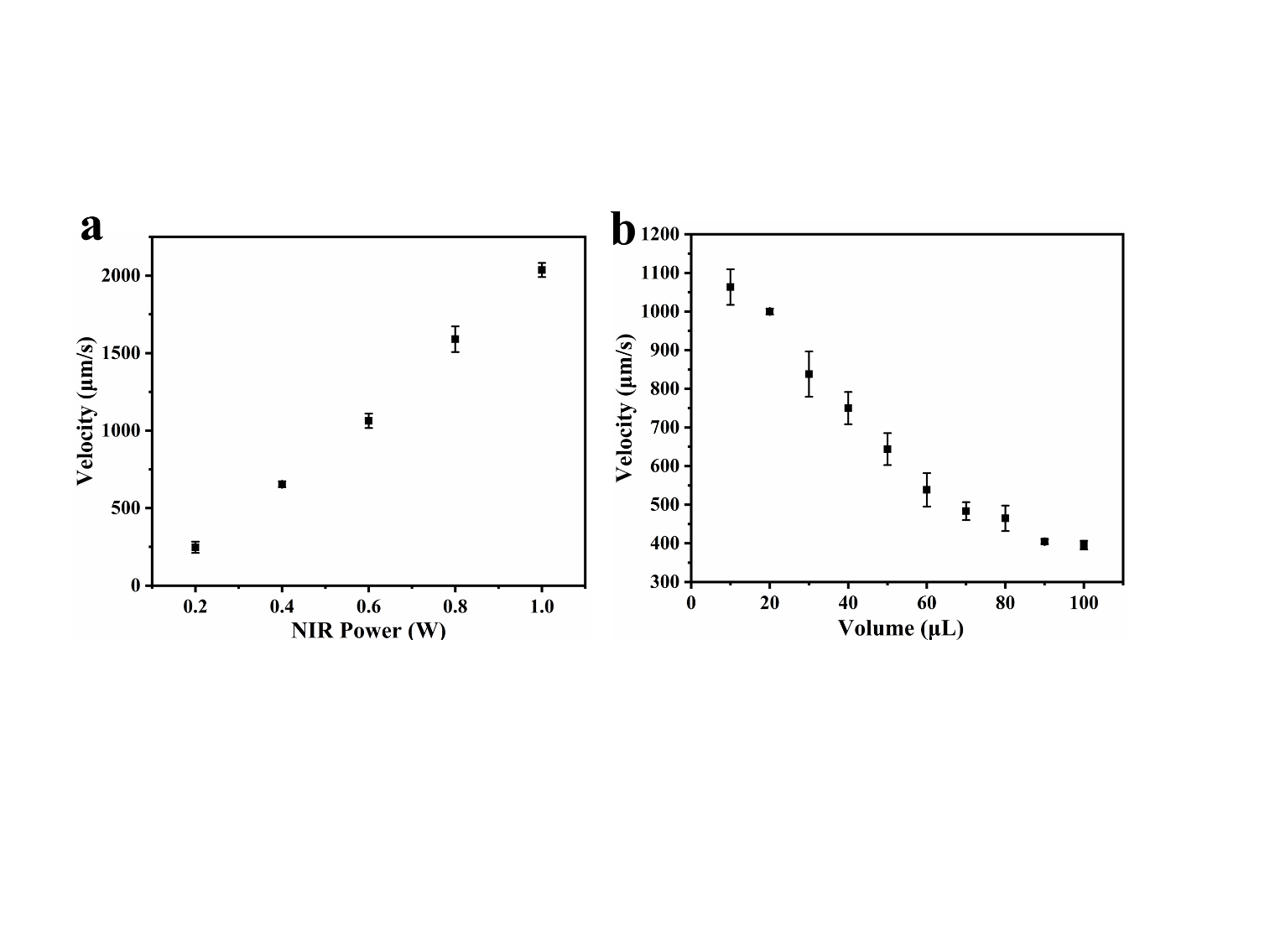


**Figure S5.** Effects of NIR power (a) and droplet volume (b) on droplet velocity.

**3. Supplementary Table**

**Table S1.** Comparison of the static delayed icing time with other studies.

| Reference | Temperature (℃) | Static delayed icing time (s) |
| --- | --- | --- |
| ^[1]^ | −18 | 165 |
| ^[2]^ | −16 | 213 |
| ^[3]^ | −15 | 192 |
| ^[4]^ | −15 | 152 |
| ^[5]^ | −10 | 78 |
| ^[6]^ | −30 | 66 |
| ^[7]^ | −30 | 82 |
| ^[8]^ | −30 | 108 |
| This work | −20 / −30 | 216 / 107 |

**Supporting References:**

[1] D. Li, L. Ma, B. Zhang, S. Chen, *Chem. Eng. J.* **2022**, *450*, 138429.

[2] Y. Tian, Y. Xu, Z. Zhu, Y. Liu, J. Xie, B. Zhang, H. Zhang, Q. Zhang, *Colloids Surf., A* **2022**, *651*, 129586.

[3] X. Li, Y. Zhao, H. Li, X. Yuan, *Appl. Surf. Sci.* **2014**, *316*, 222.

[4] Y. Liu, Y. Shao, Y. Wang, J. Wang, *Colloids Surf., A* **2022**, *648*, 129335.

[5] L. Ma, J. Wang, F. Zhao, D. Wu, Y. Huang, D. Zhang, Z. Zhang, W. Fu, X. Li, Y. Fan, *Compos. Sci. Technol.* **2019**, *181*, 107696.

[6] G. Jiang, L. Chen, S. Zhang, H. Huang, *ACS Appl. Mater. Interfaces* **2018**, *10*, 36505.

[7] H. Xie, W. Xu, C. Fang, T. Wu, *Soft Matter* **2021**, *17*, 1901.

[8] D. Wei, J. Wang, S. Li, D. Wang, Y. Liu, *Chem. Eng. J.* **2023**, *475*, 146113.

**4. Supplementary Movies**

***Movie S1***

Continuous release of droplets on supercooled SHS, S-PS and abraded S-PS surfaces.

***Movie S2***

Ice droplet movement on the S-PS and abraded S-PS by NIR actuation.

***Movie S3***

100 μL droplet movement on the S-PS by NIR actuation.

***Movie S4***

Programmable droplet manipulations on S-PS.

***Movie S5***

NIR-induced antigravity motion and coalescence of droplets on S-PS.

***Movie S6***

Droplet manipulations on the abraded S-PS.
